# Predicting Outcomes in Esophageal Squamous Cell Carcinoma Using scRNA‐Seq and Bulk RNA‐Seq: A Model Development and Validation Study

**DOI:** 10.1002/cam4.70617

**Published:** 2025-01-22

**Authors:** Jiaqi Zhang, Shunzhe Song, Yuqing Li, Aixia Gong

**Affiliations:** ^1^ Department of Digestive Endoscopy The First Affiliated Hospital of Dalian Medical University Dalian Liaoning People's Republic of China; ^2^ Department of Obstetrics and Gynecology The First Affiliated Hospital of Dalian Medical University Dalian Liaoning People's Republic of China; ^3^ Department of Gastroenterology The First Affiliated Hospital of Dalian Medical University Dalian Liaoning People's Republic of China

**Keywords:** bioinformatics, ESCC, glucose metabolism, immune infiltration, prognosis

## Abstract

**Background:**

Altered glucose metabolism is a critical characteristic from the beginning stage of esophageal squamous cell carcinoma (ESCC), and the phenomenon is presented as a pink‐color sign under endoscopy after iodine staining. Therefore, calculating the metabolic score based on the glucose metabolic gene sets may bring some novel insights, enabling the prediction of prognosis and the identification of treatment choices for ESCC.

**Methods:**

A total of 8, 99, and 140 individuals from The Gene Expression Omnibus database, The Cancer Genome Atlas database, and the Memorial Sloan Kettering Cancer Center, respectively, were encompassed in the investigation. Patients diagnosed with ESCC after surgery were enrolled for further validation.

**Results:**

A total of 13 kinds of cell clusters were screened, and the squamous epithelium was identified with the highest score. And 558 differential genes were selected from the single‐cell RNA sequencing (scRNA‐seq) dataset. Four glucose metabolism‐related genes, namely, SERP1, CTSC, RAP2B, and SSR4, were identified as hub genes to develop a risk prognostic model. The model was validated in another external cohort. According to the risk score (RS) determined by the model, the patients were categorized into low‐ and high‐risk groups (LRG and HRG). Compared with LRG, HRG indicated poor survival and decreased drug sensitivity. Additionally, the immune microenvironment and pathway enrichment were different between the two groups. Immunohistochemical staining revealed that hub genes were expressed differently in ESCC tissues, high‐ and low‐grade intraepithelial neoplasia, and adjacent normal tissues.

**Conclusion:**

Four hub genes (SERP1, CTSC, RAP2B, and SSR4) screened based on glucose metabolism developed a predictive model in ESCC patients. The RS was established as an independent risk factor for predicting prognosis. These findings may enhance understanding of ESCC's molecular profile and serve as a new prognostic tool for better patient stratification and treatment planning in clinical practice.

## 1 Introduction

Esophageal carcinoma (ESCA) is a very common and aggressive form of cancer that is responsible for a significant number of cancer‐related deaths, ranking sixth among the top causes of cancer mortality globally [1, 2]. More than 90% of all ESCA are esophageal squamous cell carcinomas (ESCC), the most common histological subtype, with a relevant high incidence in eastern Asia [3]. Various strategies have been used for the treatment of ESCC, such as surgery, endoscopic resection, and chemoradiotherapy [4]. However, formulating therapeutic strategies in clinical settings remains challenging due to the worse prognosis and low survival, with a 5‐year survival of ESCC patients still as low as 21% [5, 6]. Compared with other solid tumors such as liver cancer, the availability of pharmacological agents for immunotherapy and targeted therapy for ESCC is currently limited, with an unsatisfactory prognosis. Unfortunately, therapies with immune checkpoint inhibitors are beneficial for merely 20%–30% of ESCC patients [7]. The fundamental issue is our insufficient understanding of the molecular mechanisms underlying ESCC, particularly those related to tumor microenvironment (TME), which restraints immune cells' viability and antitumor capabilities [8]. Therefore, it is urgently needed to explore effective molecular targets for ESCC treatment, with immunotherapy providing promising and effective novel avenues [9, 10].

Altered glucose metabolism is a critical characteristic before ESCC begins [11]. Normally, massive glycogen granules exist in esophageal squamous epithelium, especially in the prickle cell layer above the basal cell layer. In epithelial tissues characterized by malignancy or dysplasia, the glycogen‐producing cells within the prickle cell layer are replaced by neoplastic cells [12, 13]. Iodine staining is essential for endoscopists to screen for precancerous lesions and early cancers. After staining, early ESCC may appear as a pink‐color sign, and even low‐grade intraepithelial neoplasia (LGIEN) may appear pale brown [13, 14]. Currently, endoscopists use this method to detect early esophageal cancer clinically. Curiously, Otto Warburg first elucidated the metabolic foundation of this occurrence in the 20th century. He observed that cancer cells engage in glycolysis, even when there is an adequate supply of oxygen [15, 16]. It is suggested that during tumor aerobic glycolysis, large amounts of lactic acid are produced, affecting the microenvironment's acidity. A PH of 6–6.9 is formed in the TME as a result of lactate and H+ released from tumor cells, which contribute to tumor growth, angiogenesis, and immunosuppression [17]. Multiple investigations have manifested the function of glucose metabolism‐related genes in ESCC development [12, 18, 19]. However, the relationship between glucose metabolism and immunological activities of ESCC has not yet been demonstrated explicitly. Therefore, decoding the complex molecular pathways related to glucose metabolism is a crucial point in exploring the impact on TME and immunotherapy of ESCC. The present study aims to develop a prognostic model for ESCC from targets related to glucose metabolism and explore its impact on TME and immunotherapy.

Conventional bulk RNA sequencing (bulk RNA‐seq) is capable of generating sufficient gene profiles for relatively large tissue specimens, yet it fails to differentiate cell lineages and their interactions effectively [20]. The advent of single‐cell RNA sequencing (scRNA‐seq) technology holds significant promise for bridging the gap between traditional high‐throughput sequencing techniques and microarray data, which compensates for the insufficiency of bulk sequencing [21]. Specifically, scRNA‐seq promotes the discovery of metabolic pathways in individual cells at the transcriptional level. That is to say, this technique allows for identifying cell subtypes that are most significantly linked to glucose metabolism changes in ESCC from mass cell types, and further analysis of these subtypes contributes to more accurate and effective prognostic features. Given these advantages, integrating bulk RNA‐seq and scRNA‐seq analyses to study tumor development and heterogeneity enables prestratification and identification of ESCC patients.

Our investigation employed data from The Cancer Genome Atlas (TCGA) and the Gene Expression Omnibus (GEO) databases to ascertain the ESCC cell types that have the strongest connections to glycolytic metabolism. Second, a glycolysis score is applied to quantify glucose metabolism, and the marker genes of the cell with relatively higher scores are characterized. Subsequently, glucose metabolism‐based risk signatures combined with clinical characteristics are utilized to create a prognostic model for ESCC patients to anticipate their survival expectations. Moreover, the high‐score gene markers are further explored in the aspect of immune activities, drug sensitivity, correlated pathways, transcription factor function prediction, etc. These explorations provide a broader perspective on biological processes, allow for more effective therapeutic strategies, and offer personalized treatment for ESCC patients.

## 2 Materials and Methods

### 2.1 Data Sources Download

The National Center for Biotechnology Information (NCBI) is in charge of developing and maintaining the GEO database, which may be accessed at https://www.ncbi.nlm.nih.gov/geo/info/datasets.html. Data files for GSE188900 were obtained from the NCBI GEO public repository, including single‐cell expression profiles for eight case samples used in the analysis. Ninety‐nine ESCC patients' processed raw expression data and clinical information were downloaded using the R package TCGAbiolinks from the TCGA database (https://cancergenome.nih.gov/). Clinical samples from the MSKCC Biobank, containing expression profile data of 140 patients, were utilized in this research study (https://www.cbioportal.org/). Gene sets related to glucose metabolism were obtained from the Molecular Signature Database (MSigDB) (https://www.gsea‐msigdb.org/gsea/msigdb) and relevant literature sources [22, 23, 24]. Three hundred twenty‐two related genes were used for the subsequent analysis after eliminating genes that overlapped.

### 2.2 Data Processing of ScRNA‐Seq Data and Identification of DEGs of ESCC

Firstly, the R package “Seurat” was employed to screen out the abnormal expression samples. In this investigation, the data specimens were first filtered employing gene numbers (nFeature_RNA), sequencing depth (nCount_RNA), and mitochondrial gene percentage (percent.mt) to demonstrate the reliability of the data. The setting parameters are as follows: nFeature_RNA > 200 & nFeature_RNA < 7500 & percent.mt < 15 & nCount_RNA < 50,000. After standardizing and normalizing the data, a principal component analysis (PCA) was executed to reduce noise, followed by using a scree plot to identify the optimal number of principal components. By employing uniform manifold approximation and projection (UMAP) for nonlinear dimensionality reduction [25], the spatial arrangement of each cluster was determined. For cell type annotation, we utilized the R package “Seurat,” in conjunction with the CellMarker database [26] and the PanglaoDB database [27]. Additionally, utilizing the glucose metabolic gene sets, the metabolic score of each specimen in the scRNA‐seq dataset was computed employing the single‐sample gene set enrichment analysis (ssGSEA) approach. The metabolic level of each subtype of cell was reflected by ssGSEA scores. After extracting gene expression files of the squamous epithelium, the filtering conditions were logFCfilter > 1 & adjPvalFilter < 0.05 for finding differentially expressed genes (DEGs).

### 2.3 Development and Testing of a Predictive Model

Following the elimination of prognostic genes through univariate Cox regression, a predictive‐linked model was then constructed employing the least absolute shrinkage and selection operator (LASSO) regression analysis [28] with the R package glmnet. The weights of the risk score (RS) model were derived using the levels of a gene expressed by each optimal prognosis (RS = ∑𝑛𝑛=1𝑐𝑜𝑒𝑓𝑖*𝑥𝑖.). In short, the expression of each lasso gene is multiplied by its corresponding LASSO coefficient. Patients were classified into low‐risk group (LRG) and high‐risk group (HRG) depending on their RS, and the threshold for determining high risk was set at the median RS value. The Kaplan–Meier approach was applied to ascertain variations in survival rates between the two groups, and these distinctions were further analyzed employing the log‐rank test. The receiver operating characteristic (ROC) curve is employed to ascertain the precision of model predictions in ESCC patients [29]. Ultimately, we utilized the MSKCC datasets as the outside validation cohorts.

### 2.4 Analysis of RS and Clinical Characteristics

ESCC patients were classified into subgroups according to clinicopathological characteristics, encompassing survival status, gender, T, M, N, and clinical stages. The Kruskal–Wallis or the Wilcoxon rank test was applied to ascertain the connection between the RS and clinical variables, as well as the variations in RS level among different clinical characteristic subtypes. Both multivariate and univariate Cox regressions were performed to determine if the RS could independently anticipate clinical outcomes.

### 2.5 Analysis of Immune Cell Infiltration and Immune Regulators

The CIBERSORT deconvolution method was applied to estimate the presence of immune cells [30], which includes 547 markers that differentiate 22 various human immune cell types like T cells, B cells, myeloid cell subsets, and plasma cells. The investigation utilized the CIBERSORT method to examine the data of patients with ESCC in order to ascertain the relative distribution of 22 types of immune‐infiltrating cells and compare the changes in immune cells among different risk categories. To conduct a more thorough examination of the disparities between RSs and immune regulators, TISIDB, an immune‐related database combining a total of 988 immune antitumor genes from seven databases, was explored [31].

### 2.6 Drug Sensitivity Analysis

Utilizing the extensive pharmacogenomics repository from the Genomics of Drug Sensitivity in Cancer (GDSC) at https://www.cancerrxgene.org/, we employed the R package “pRRophetic” to forecast the responsiveness of individual tumor samples to chemotherapy. Through regression analysis, we determined the estimated half‐inhibitory concentration (IC50) for each particular chemotherapy drug. Subsequently, we validated the accuracy of the regression and predictions by conducting a 10‐fold cross‐validation on the GDSC training set. All settings, including “combat” for eliminating batch effects and calculating the mean of duplicate gene expression, were set to their default levels.

### 2.7 Gene Set Variation Analysis (GSVA)

GSVA is a nonparametric, unsupervised method for determining gene set enrichment in the transcriptome [32]. GSVA is a method that transforms alterations in individual genes into changes in whole pathways by thoroughly examining a specific gene set and then ascertaining the biological role of the specimen. In the R package “GSVA,” enrichment scores were calculated from samples from the LRG and HRG. The gene sets are obtained from the MsigDB database and evaluated thoroughly by employing the GSVA method to ascertain any alterations in biological function across different specimens.

### 2.8 Gene Set Enrichment Analysis (GSEA)

GSEA is a commonly applied technique for assessing whether a gene set displays significant changes between two biological traits. Patients were categorized into HRG and LRG depending on the RS, followed by GSEA analysis to find the variations in signaling pathways between the groups. Pathway analysis is conducted to compare gene expression differences among different biological phenotypes [33]. Gene sets that are significantly enriched are identified and then organized according to their consistency score, with a threshold of *p* < 0.05.

### 2.9 Transcription Factor Prediction

The research applied the R package “RcisTarget” to forecast transcription factors using only motif‐based computations. An enrichment score's normalized enrichment (NES) is influenced by the overall number of motifs within its database. In addition to the motifs found in the original data, this study developed additional annotation files by taking into account motif similarity and gene sequences. To ascertain the overexpression degree for each motif within a gene set, the first step consisted of computing the area under the curve (AUC) for each pair of motif and gene. This analysis was conducted utilizing recovery curve calculations to compare the gene set with the sequencing of the motifs. The NES was computed for each motif by assessing the AUC of all motif distributions within the gene set.

### 2.10 Nomogram Prediction Model Construction

The nomogram was applied as a precise quantitative instrument to forecast the prognosis of an individual patient based on their RS and clinical symptoms. It utilized line segments with scales on a single plane to depict the relationship between variables in the prediction model [34]. A multivariate regression model is employed to give a numerical score to each value level of every variable, taking into account its influence on the result variable (the regression coefficient). These individual scores are then combined to calculate the total score to predict the value. Then, validation of the nomogram was based on calibration, ROC, and decision curve analysis.

### 2.11 Study Approval

The current investigation adhered to the guidelines outlined in the Helsinki Declaration. Approval for the investigation was granted by the Ethics Committee of the First Affiliated Hospital of Dalian Medical University under the number PJ‐KS‐KY‐2024‐406.

### 2.12 Immunohistochemistry (IHC) Staining

We received a total of 16 pairs of ESCC tissues and their matched normal esophageal tissues from the Department of Pathology at the First Affiliated Hospital of Dalian Medical University. Additionally, we obtained eight tissues with high‐grade intraepithelial neoplasia (HGIEN) and 12 tissues with LGIEN for immunochemical analyses. IHC staining was conducted with the assistance of Servicebio (Wuhan, China). The primary antibodies used in this study include an anti‐SERP1 antibody from Proteintech (17807‐1‐AP), an anti‐CTSC antibody from Abcam (ab314644), an anti‐RAP2B antibody from Bioss (bs‐2330R), and anti‐SSR4 antibody from Proteintech (11655‐2‐AP). The images were obtained with a Nikon Digital Sight DS‐Fi2 digital camera and Nikon NIS Elements D Software version 4.20, with magnifications of 200x and 400x.

### 2.13 Statistical Analysis

The aforementioned studies were applied employing the R program (Version 4.0.3). Prognostic genes were identified using Cox regression analysis. Kaplan–Meier method was utilized for survival analysis. Comparison between groups was calculated using the Wilcox test. A *P*‐value *<* 0.05 was deemed to have statistical significance.

## 3 Results

**FIGURE 1:**
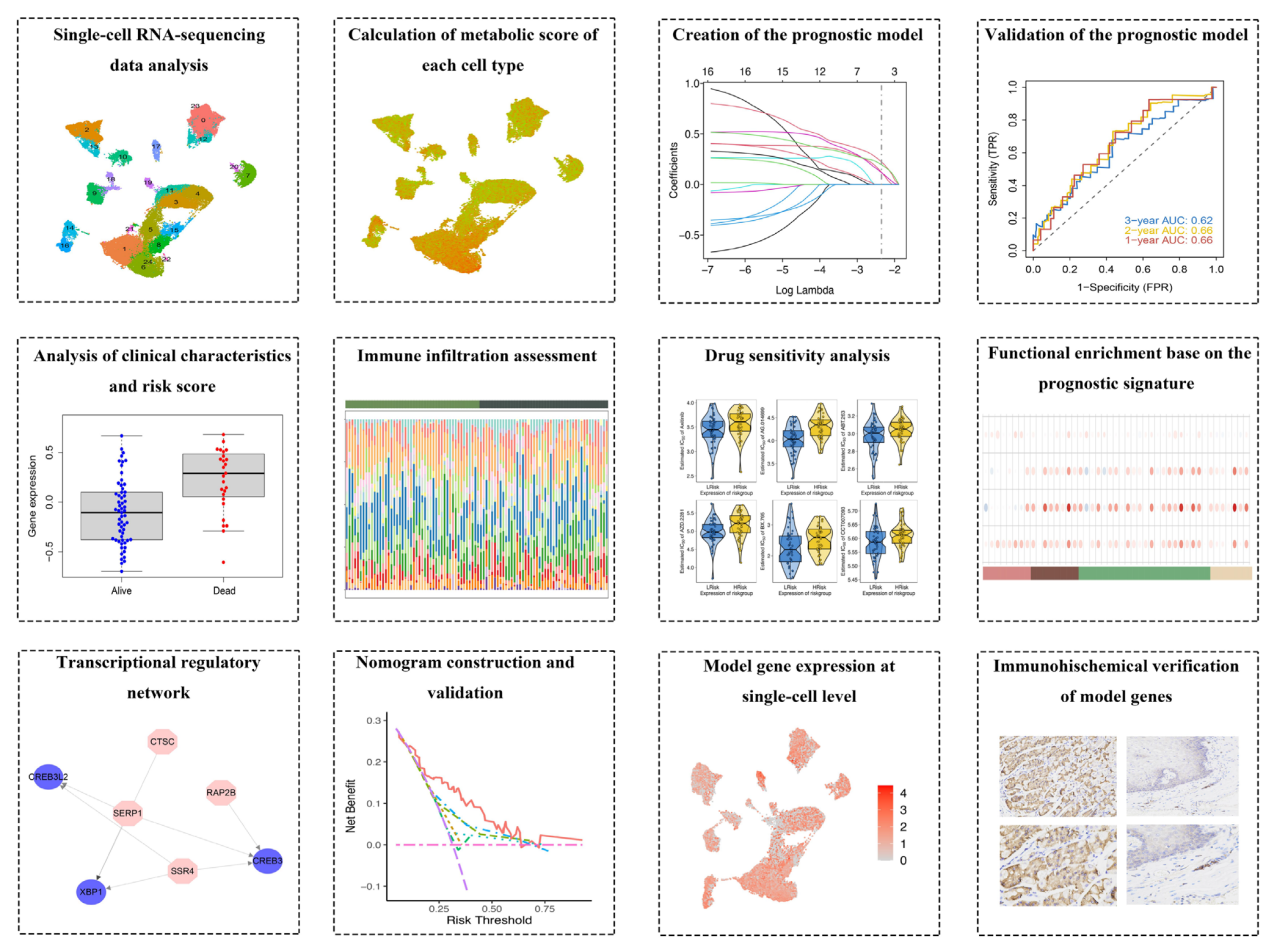
Schematic diagram illustrating the overall study design.

A schematic flow of our study is shown in Figure 1.

### 3.1 Identification of Cell Subtypes of ESCC

**FIGURE 2:**
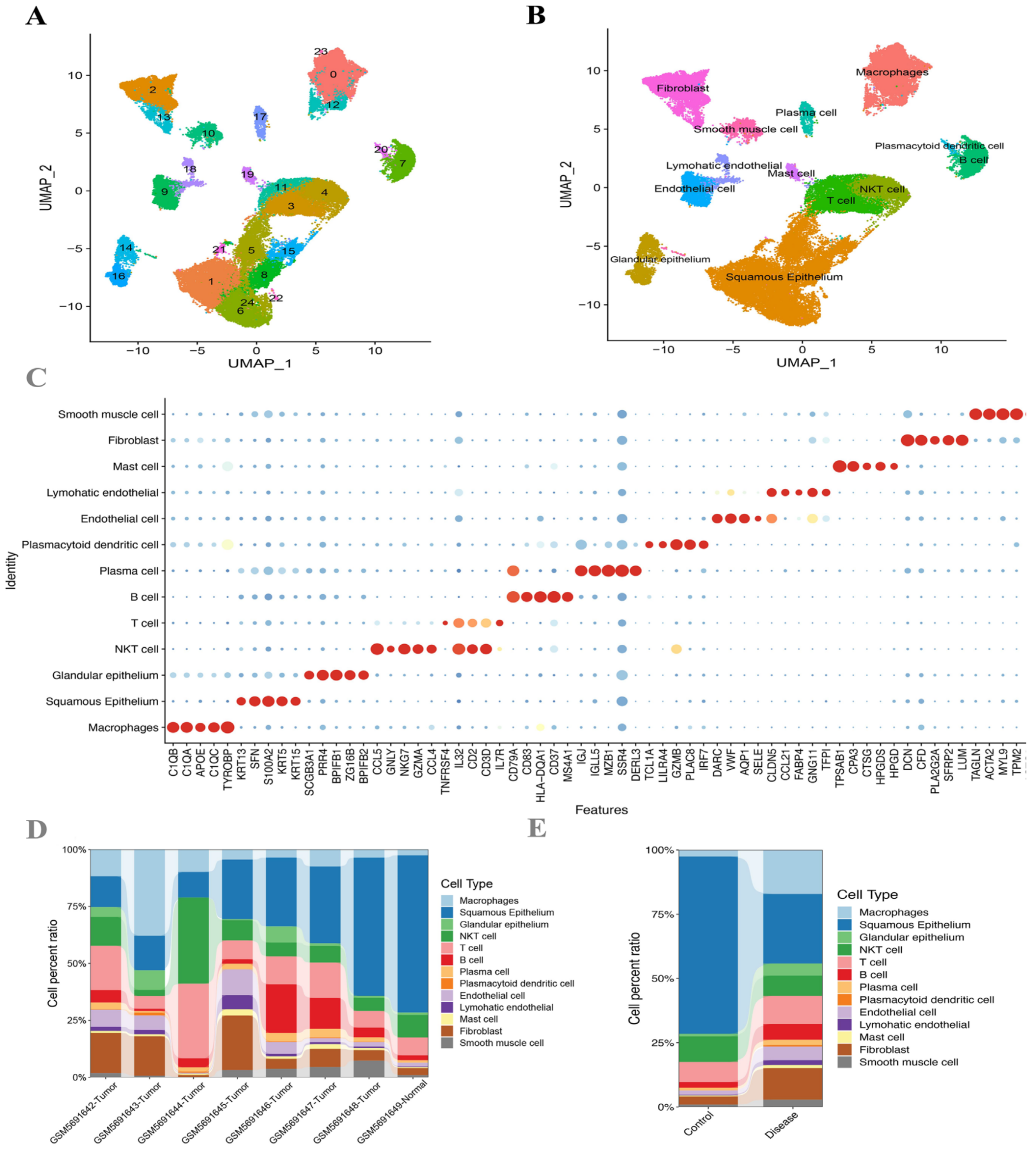
Clustering and annotation of the ESCC cells. (A) UMAP plot of 51,134 cells was divided into 25 clusters. (B) The 25 cell clusters were annotated into 13 major types using CellMarker and PanglaoDB. (C) The bubble chart shows specific markers corresponding to 13 different cell types. (D) Variations in the content percentages of 13 cell types in 8 groups of samples. (E) Cell distributions differ between individual samples in control and disease groups.

**FIGURE 3:**
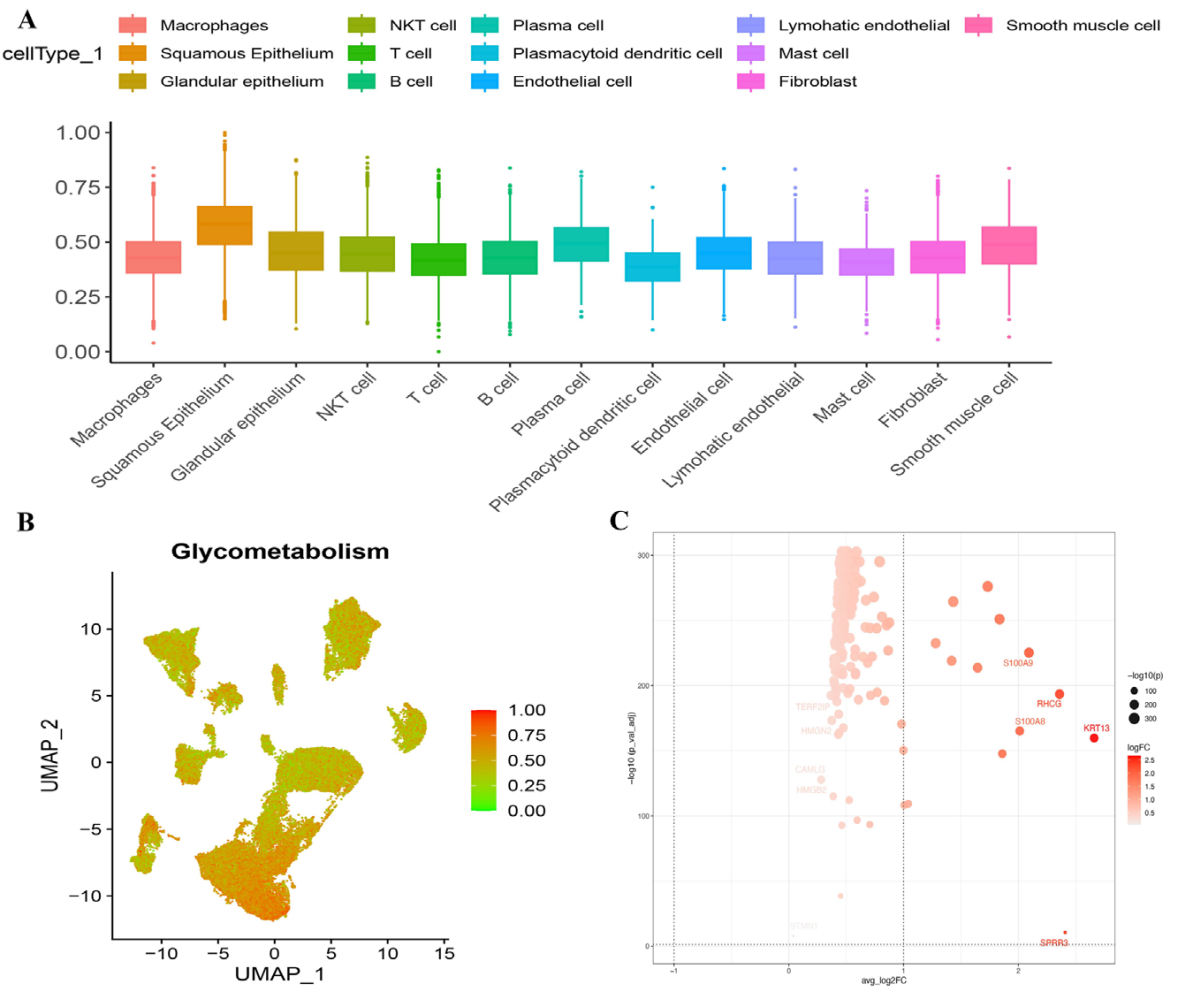
Expression differences in glycosylation scores. (A, B) Expression profiles of marker genes were obtained among the 13 cell types in ESCC by calculating the ssGSEA score of each cluster. (C) A Volcano plot exhibited DEGs of squamous epithelium cells.

A total of 51,134 cells were derived (Figure S1A,B), and we extracted the 10 genes that have the largest standard deviations (Figure S1C). The data underwent standardization, homogenization, PCA, and harmony analysis in sequence (Figure S1D–F). UMAP analysis then identified 25 cell clusters (Figure 2A), and each subtype was further annotated to macrophages, squamous epithelium, glandular epithelium, NKT cell, T cell, B cell, plasma cell, plasmacytoid dendritic cell, endothelial cell, lymphatic endothelial, mast cell, fibroblast, and smooth muscle cells—13 cell types in total (Figure 2B). The bubble chart of the classic markers of 13 types of cells (Figure 2C), the histogram of cell proportions corresponding to each sample (Figure 2D), and the histogram of cell proportions corresponding to the group (Figure 2E) are as shown. According to the expression of glucose metabolic genes, the squamous epithelium was found to have the highest score (Figure 3A,B) among 13 cell types, and a total of 558 DEGs were screened out (Figure 3C).

### 3.2 Construction and Validation of a Four‐Characteristic Gene‐Based Prognostic Model

**FIGURE 4:**
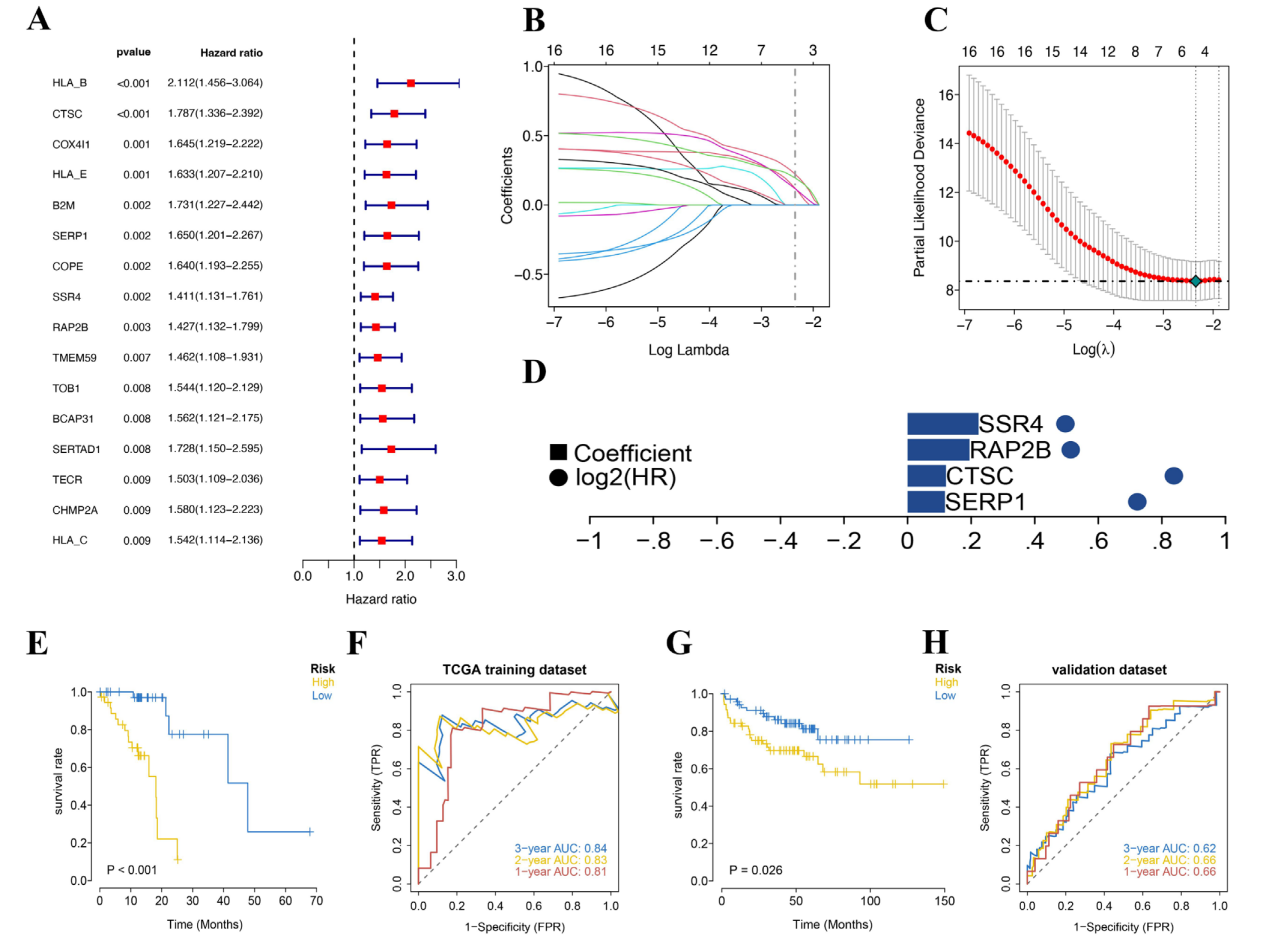
Creation and validation of the prognostic model. (A) Forest plot of prognostic significant glucose metabolism‐related genes. (B, C) Lamba curves and cvfit showing the LASSO regression were performed with the minimum criteria. (D) The distribution of LASSO coefficients for gene combinations and prognosis‐related genes at the minimal lambda value. (E) The Kaplan–Meier survival curve of ESCC for the internal training set in the TCGA cohort (*p* < 0.001). (F) ROC curves for TCGA (1, 2, and 3 years). (G) The Kaplan–Meier survival curve of ESCC for the external testing set in the MSKCC cohort (*p* = 0.026). (H) ROC curves for MSKCC (1, 2, and 3 years).

The clinical data of ESCC patients were acquired from the TCGA database. Differential genes associated with glucose metabolism were used to identify predictive genes in ESCC by univariate Cox regression analysis. The outcomes manifested that a collective of 17 genes (*p* < 0.01) had a significant connection with overall survival (OS) (Figure 4A). Then, the processed ESCC dataset was randomly assigned in the TCGA database with survival data into training and testing sets at a ratio of 4:1, and each sample was obtained through LASSO regression analysis (Figure 4B,C). Univariate Cox regression analysis was conducted using the LASSO Cox regression model to avoid excessive model fitting, and four genes (SERP1, CTSC, RAP2B, and SSR4) were selected as specific genes for subsequent model construction (Figure 4D). The corresponding optimal RS value is used for subsequent analysis RS = SERP1 expression level x 0.117391097872757 + CTSC expression level × 0.120528949130514 + RAP2B expression level × 0.194582426695387 + SSR4 expression level × 0.223000735053101. The patients were classified into HRG and LRG depending on their risk ratings and then examined with Kaplan–Meier curves. The HRG survival rate was significantly mitigated contrasted with the LRG in both the training (*p* < 0.001) and the testing sets (*p* = 0.01) (Figure 4E). The ROC curves for both datasets manifest that the model has a robust performance in terms of verification (Figure 4F). Furthermore, the model was externally validated using data from 140 ESCC patients in the MSKCC cohort. The outcomes manifested that the HRG had a significantly mitigated survival rate (*p* = 0.026) contrasted with the LRG (Figure 4G). Additionally, the model emerged with a robust ability to anticipate the ESCC patient's prognosis (Figure 4H).

### 3.3 Analysis of the Distribution of RSs in Different Clinical Features

**FIGURE 5:**
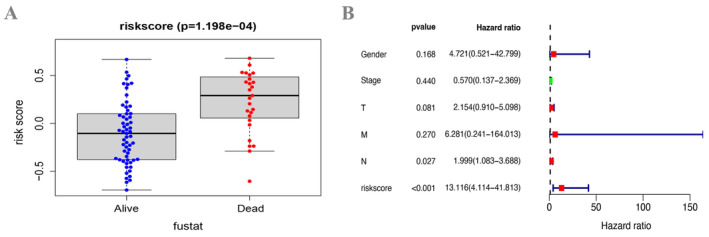
Multivariate Cox analysis of RS and clinical correlation analysis. (A) Association of RS and clinical characteristics (survival status) of ESCC patients. (B) Cox regression (multivariate) manifested that the RS was an autonomous risk factor *(p* < 0.001) in ESCC patients.

We allocated the specimens depending on the RS values by comparing the clinical indicator values. The findings are shown in a box plot format (Figures 5A and S2A–E). The statistical analysis revealed a significant variation (*p* < 0.001) in the distribution of RS values across groups based on survival status (Figure 5A). However, there were no statistical differences regarding other factors such as M (*p* = 0.564), N (*p* = 0.357), clinical (*p* = 0.603), T stages (*p* = 0.787), and gender (*p* = 0.074) (Figure S2A–E). Furthermore, the RS was shown to be a significant separate predictive factor (*p* < 0.001, *p* < 0.001, respectively) for ESCC patients, as demonstrated by both univariate and multivariate Cox regression analyses (Figures 5B and S2F).

### 3.4 Hub Genes Connected With Immune Infiltration and Immune Regulators

The CIBERSORT deconvolution algorithm transformed normalized FPKM gene expression data into immune cell information (both cell types and cell proportions). Specifically, after obtaining the FPKM values of each expressed gene from ESCC bulk‐RNA seq, 22 immune cell subsets in ESCC were evaluated using the CIBERSORT algorithm. The information of 22 cells is shown in Figure S3A, which cannot be acquired from only bulk‐RNA seq. Then, the CIBERSORT algorithm could characterize the cell composition (both cell types and cell proportions) of each sample. Figure S3A displayed the immune cell content distribution in the two genetic expression subtypes (low or high expression level), and Figure S3B showed the difference in immune cell content between HRGs and LRGs. Specifically, the contents of eosinophils and M2 macrophages were significantly (*p* < 0.05, *p* < 0.05, respectively) different between high‐ and low‐ expression subtypes, suggesting that these cells may play a key role in the immune microenvironment in different risk groups (Figure S3B). Figures S3C and S4 further demonstrated the differences in the expression of different categories of immune factors between HRGs and LRGs. Through obtaining immune regulatory genes from the TISIDB database, including immune stimulatory factors, immunosuppressive factors, MHC genes, chemokines, and their receptors, the expression patterns of these factors were analyzed. The results showed that the expression of multiple immune factors was significantly different between HRGs and LRGs. In terms of immune function, the results revealed that the expression of CD86‐, KIR2DL1‐, and MHC‐related genes, CCR3, CCL18, CCL20, and CCL4, was upregulated in the HRG (Figures S3C and S4A–D), while the expression of CCR10 and CCL14 was upregulated in the cohort exhibiting low risk (Figure S4C,D).

### 3.5 Drug Sensitivity, GSVA, GSEA Analysis, and Transcription Factor Prediction

**FIGURE 6:**
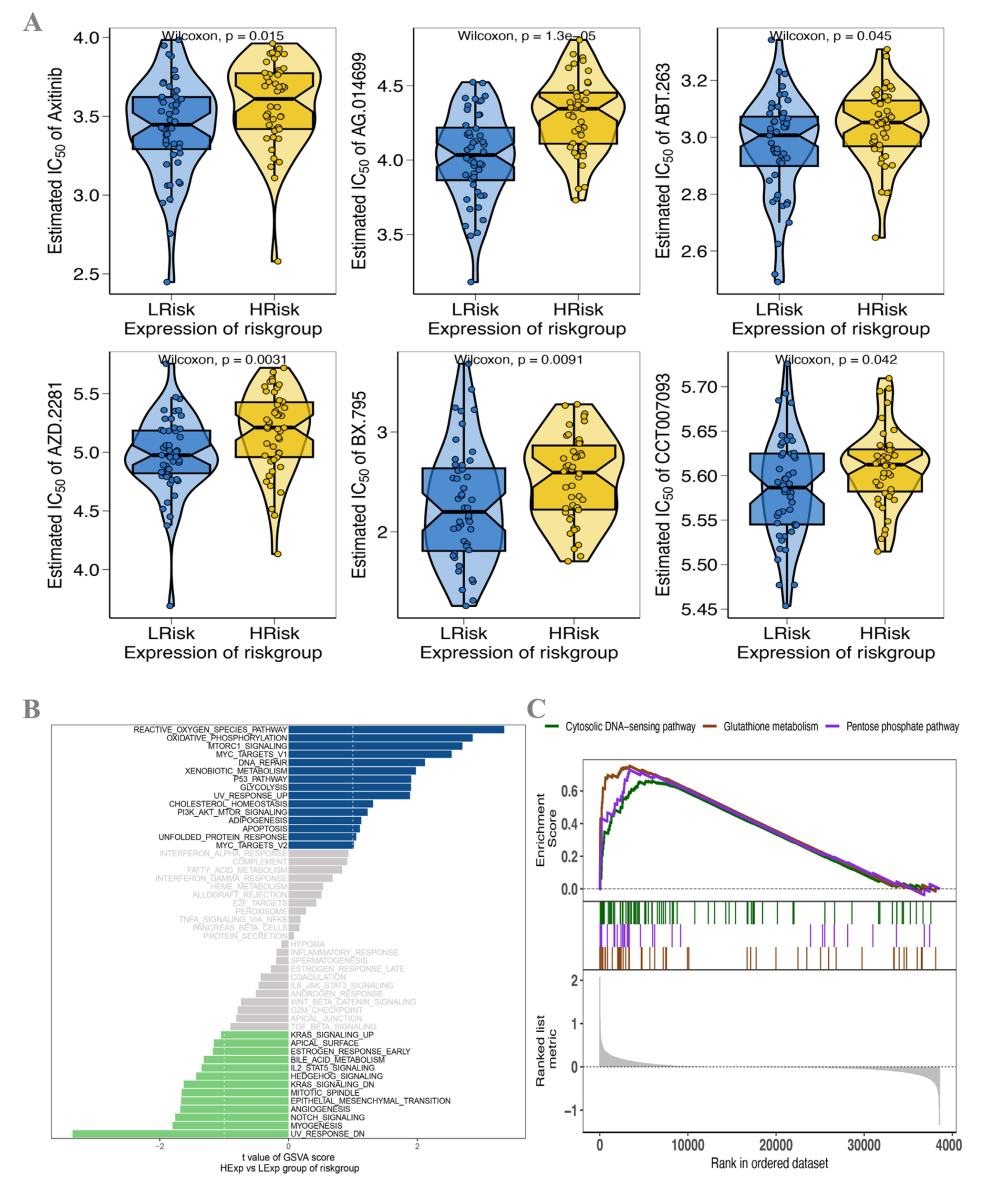
Drug susceptibility prediction and visualization of the results of GSEA and GSVA analyses. (A) Sensitivity analysis of HRG and LRG to chemotherapeutic drugs. (B) GSVA of the prognostic model genes. (C) GSEA of the prognostic model genes.

Upon executing a comparative examination of the effectiveness of various chemotherapeutic medicines across distinct clusters, we observed variations in drug sensitivity between these two groups. The RS level shows a significant connection with the sensitivity of ESCC patients to axitinib (*p* < 0.05), AG.014699 (*p* < 0.001), ABT.263 (*p* = 0.045), AZD.2281 (*p* < 0.01), BX.795 (*p* < 0.01), and CCT007093 (*p* < 0.05) (Figure 6A). Subsequently, the researchers examined the precise signaling pathways implicated in both the high‐ and low‐risk correlation models [32, 33, 35]. We also investigated the probable molecular mechanisms via which RSs influence the advancement of tumors. The GSVA analysis revealed that the distinct groups of patients had significant enrichment in pathways encompassing REACTIVE_OXYGEN_SPECIES_PATHWAY, MTORC1_SIGNALING, and P53_PATHWAY (Figure 6B). The results of GSEA showed that the pentose phosphate pathway, cytosolic DNA‐sensing pathway, and glutathione metabolism are noticeable (Figure 6C). Following that, the four hub genes were used as a gene set for this research and were discovered to be controlled via shared mechanisms, encompassing numerous transcription factors. The study of essential genes revealed that the motif with the greatest NES (7.84) is cisbp__M0340 by motif‐TF annotation and selection analysis. The research displayed all the enriched motifs and their related transcription factors of the hub genes using cytoscape (Figure S5A,B).

### 3.6 Nomogram Construction and Validation

To create a useful quantitative tool for clinical practice, we conducted a multivariate logistic regression analysis. The outcomes of this analysis were presented in the form of a nomogram. The analysis showed that the RS value is significant in the scoring process of the prediction model for all of our samples. By drawing a vertical line from the total points on the nomogram to the survival prediction axis, we were able to anticipate the 1‐ and 2‐year survival rates of each patient with ESCC (Figure S6A). The findings indicated that the OS of ESCC patients mitigated at the 1‐ and 2‐year marks as the total scores elevated. The calibration curve closely resembled the optimal curve (Figure S6B), and plotted ROC curves and DCA curves for the 1‐, 2‐, and 3‐year periods of ESCC, respectively (Figure S6C,D), which indicates that the predictive ability of the nomogram has medium accuracy.

### 3.7 Expression Situation of Hub Genes at the Single‐Cell Level

**FIGURE 7:**
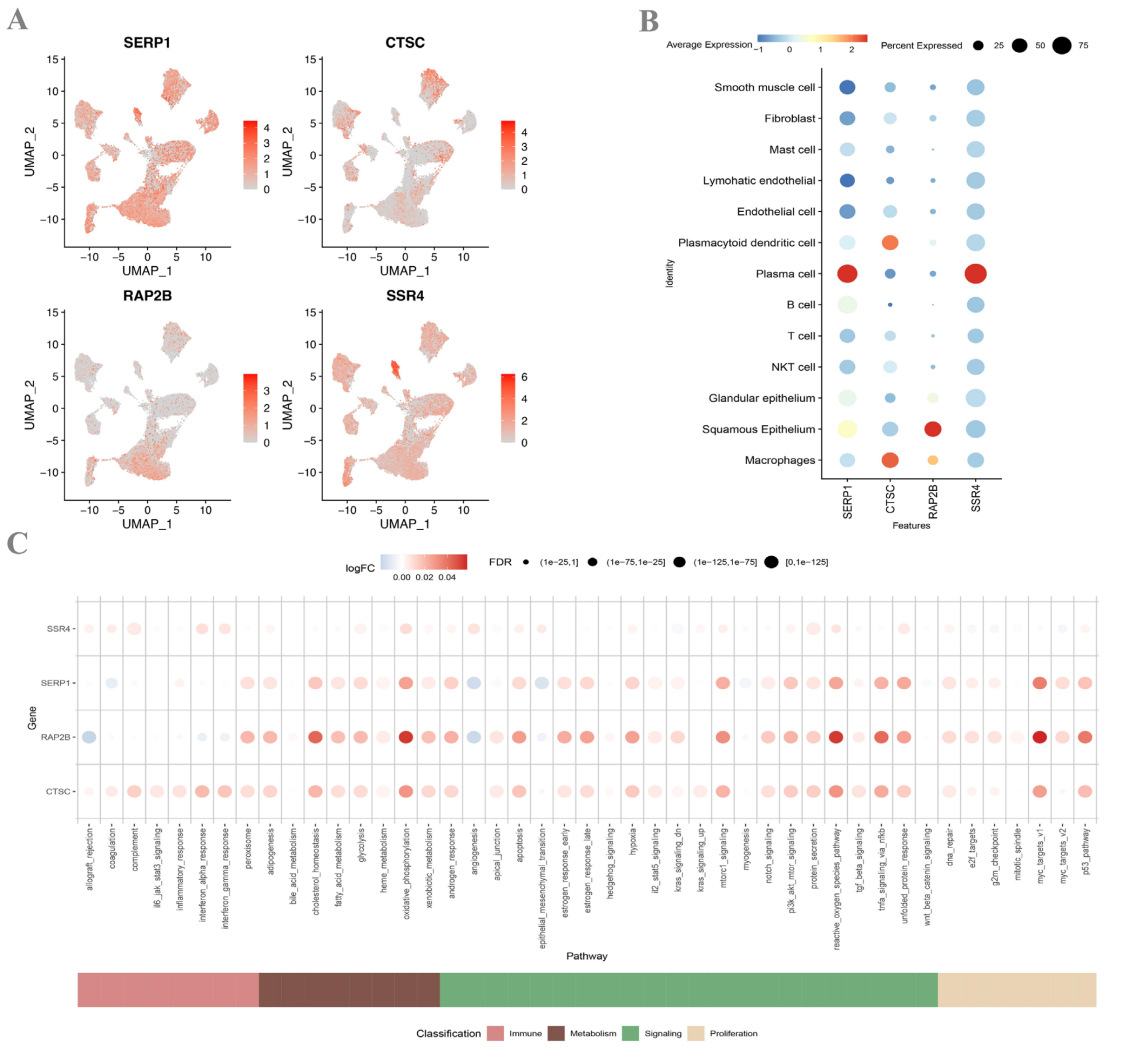
Hub genes expression at single cell level. (A, B) Single‐cell gene expression of four hub genes. (C) Connection between hub genes in predictive risk model and the immune and metabolic pathways.

The expression of four hub genes was demonstrated in 13 types of cells, and the outcomes manifested that SERP1 and SSR4 are highly expressed in plasma cells, CTSC expression is high in macrophages, and RAP2B expression is more highly expressed in squamous epithelium cells (Figure 7A,B). Finally, the AUCell function was used to perform quantitative analysis of immune and metabolic pathways on single‐cell data, depicting a signal pathway for the high and low expression of each key gene in a single cell.

The connection between hub genes and immune and metabolic pathways was enriched to multiple pathways, such as GLYCOLYSIS, OXIDATIVE _ PHOSPHORYLATION, MTORC1 _ SIGNALING, and PI3K _ AKT _ mTOR _ signaling.

### 3.8 Validation of the Levels of Hub Genes in Clinical Samples

**FIGURE 8:**
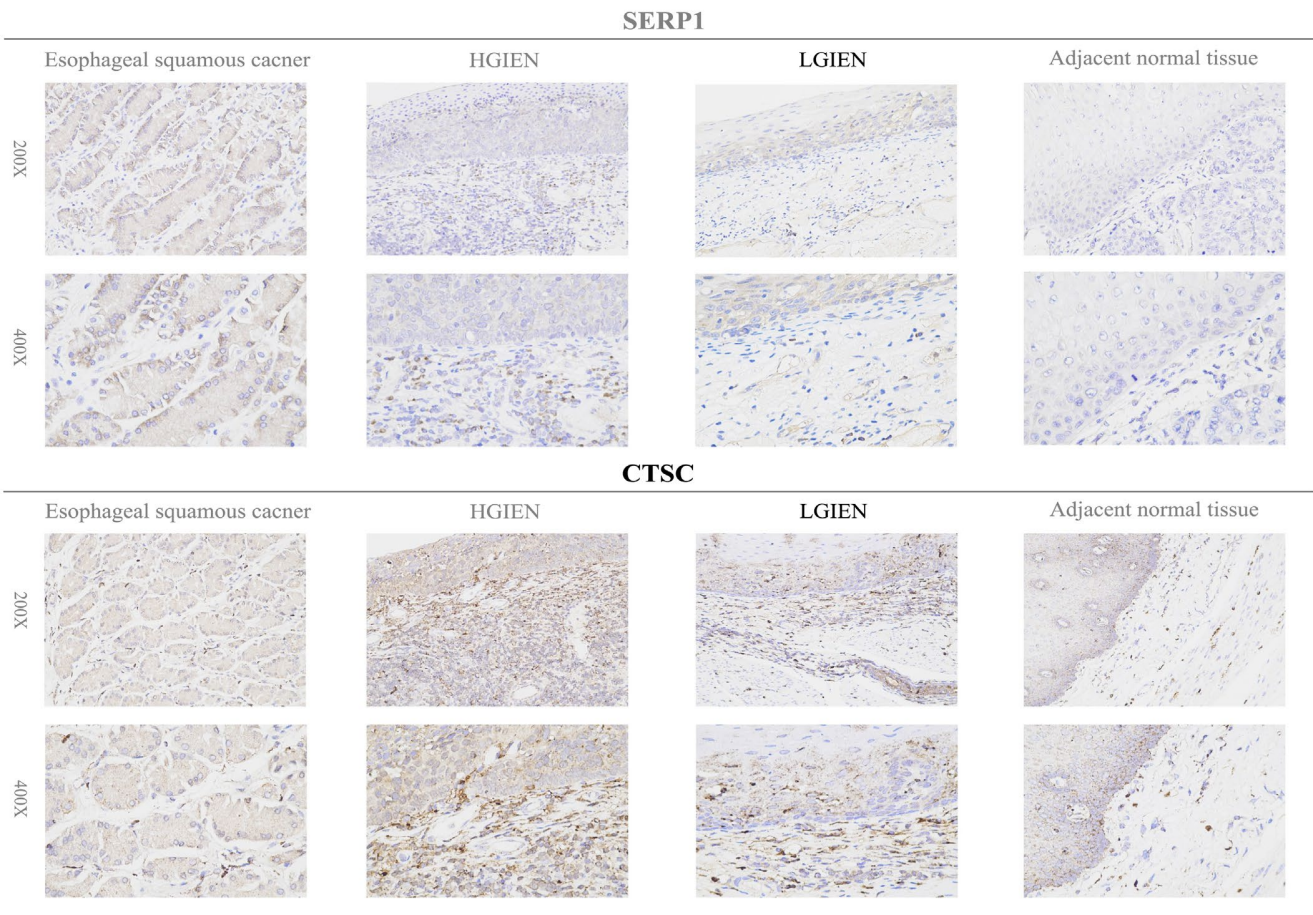
Immunohistochemical verification of prognostic genes (SERP1 and CTSC) in esophageal squamous carcinoma, HGIEN, LGIEN, and adjacent normal tissues.

**FIGURE 9:**
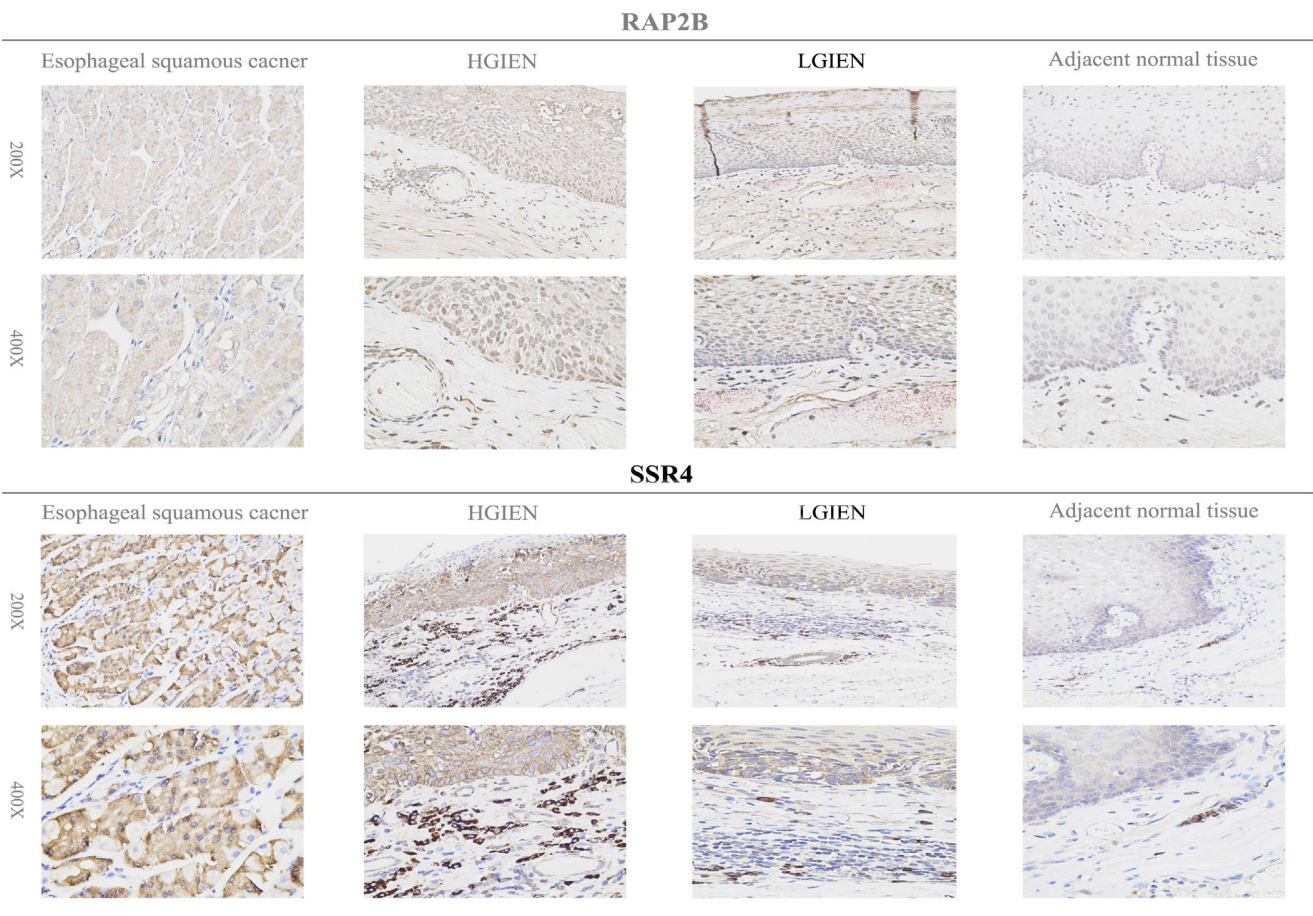
Immunohistochemical verification of prognostic genes (RAP2B and SSR4) in esophageal squamous carcinoma, HGIEN, LGIEN, and adjacent normal tissues.

**FIGURE 10:**
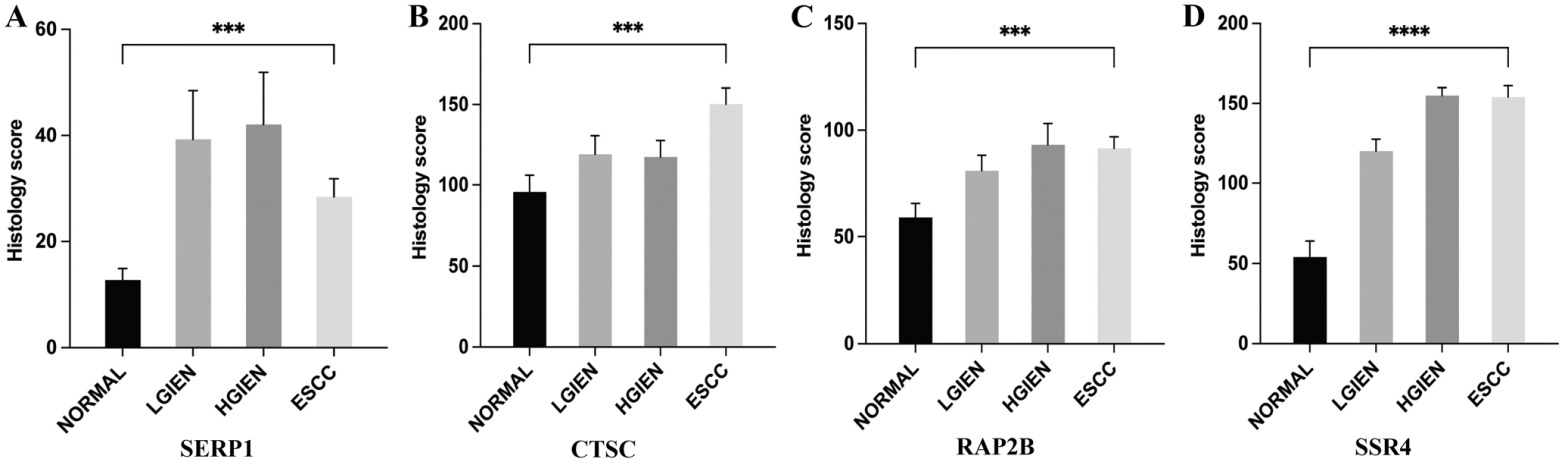
Bar graph showing the results of semiquantitative analysis of the immunohistochemistry of (A) SERP1, (B) CTSC, (C) RAP2B, and (D) SSR4 in ESCC (*n* = 16), HGIEN (*n* = 8), LGIEN (*n* = 12), and adjacent normal tissues (*n* = 16). (*p < 0.05, **p < 0.01, ***p < 0.001, ****p < 0.0001.)

The IHC data depicting the expression of SERP1, CTSC, RAP2B, and SSR4 on tissues affected by ESCC, HGIEN, LGIEN, and adjacent normal tissues are shown in Figures 8 and 9. Our outcomes manifest that the hub genes protein expression level was significantly greater in esophageal tumor tissues than in neighboring normal tissues. In addition, early ESCC patients had a range of expression levels, spanning from low to high. The varying levels of expression of hub genes may account for the intrinsic disparities in biological features seen across individuals with ESCC. Representative results of IHC staining are shown in Figures 8 and 9. Compared with adjacent normal tissues, the expression of the four genes (SERP1, CTSC, RAP2B, and SSR4) was significantly increased in ESCC patients (*p* < 0.001, *p* < 0.001, *p* < 0.001, *p* < 0.0001, respectively) (Figure 10A–D).

## 4 Discussion

Currently, iodine staining serves as a crucial diagnostic point for endoscopists in the clinical detection of early esophageal cancer. This approach is on the basis of the abnormal glucose metabolism of esophageal cancer cells. Nevertheless, few studies reported predictive models of ESCC prognosis based on glucose metabolism. In this study, glucose metabolism was identified as an entry point for investigating ESCC. An integrated analysis of scRNA‐seq data and clinical datasets was performed to identify key prognostic biomarkers and construct a robust predictive model for ESCC. After being quantified by the ssGSEA score, the squamous epithelium cells with the most effective points related to glucose metabolism were selected for subsequent analyses. Four hub genes (SERP1, CTSC, RAP2B, and SSR4) associated with glucose metabolism were identified to predict OS in ESCC patients. Both internal and external cohorts validated the high accuracy of the predictive model on the basis of the hub genes. Additionally, our analysis of immune infiltration and drug sensitivity further elucidated the potential relationship between ESCC and glucose metabolism. Ultimately, we verified the expression of hub genes in ESCC, HGIEN, LGIEN, and neighboring normal tissues employing IHC staining. These findings may offer valuable insights into the molecular landscape of ESCC and provide a novel prognostic tool that could be used in clinical practice to improve patient stratification and treatment planning.

During the initiation and progression of solid tumors, cellular metabolism is fundamentally rewired [36]. Through performing scRNA‐seq analysis of ESCC, all 51,134 cells were annotated to 13 core cellular clusters, and squamous epithelium cells were selected from the 13 cell clusters with the highest score related to glycometabolism. Then, 558 differential genes from squamous epithelium were obtained, and hub genes from the 558 genes were employed to further create a risk model to predict the ESCC patient's prognosis. Four prognosis‐associated and glucose metabolism‐related genes (SERP1, CTSC, RAP2B, and SSR4) were ascertained, with the ROC curve results indicating that it may present promising efficacy in predicting the development and outcome of ESCC in both internal and external cohorts. Furthermore, our findings showed that the patients in the HRG possessed higher mortality and a worse short‐term outcome. Among the four identified hub genes, SERP1 serves as a novel cochaperone and regulator of epithelial sodium channel levels [37], which is regarded as a major chaperone protein involved in the unfolded protein response [38]. Li et al. reported that SERP1 is involved in an ESCC prognostic prediction model based on nine endoplasmic reticulum stress mRNA signatures, and functioned as a poor tumor prognosis gene [39]. In contrast, Fan et al. found that low SERP1 expression is associated with immune cell infiltration and poor prognostic in patients with skin cutaneous melanoma [40]. In the present study, the SERP expression in HGIEN increased significantly than that in the normal tissue, but it decreased in the ESCC stage. The mechanism is worth further research. CTSC is a significant acid hydrolase found in the lysosomes [41]. Defects in CTSC‐encoded protein have been proven to be a cause of Papillon–Lefevre syndrome, which is presented as excessive keratinization [42]. This indirectly validates the relationship between CTSC and ESCC. Early ESCC may have a loss of keratinization on the surface of squamous epithelium under endoscopy [42]. And keratinization is related to the metabolism of glycosaminoglycans [43]. Additionally, CTSC has been reported to promote metastasis by regulating the recruitment of neutrophils and the formation of neutrophil extracellular traps [44]. SSR4 serves as a component of translocation‐associated protein, and its removal may result in the development of congenital abnormalities related to glycosylation [45]. It was identified as a prognostic biomarker and found to be associated with immune infiltration in colon adenocarcinoma [46]. It was suggested that RAP2B is a conserved p53‐activated gene [47], and it was also reported to be correlated closely with various cancers [48]. A research based on cuproptosis and ferroptosis also emphasized the predictive role of RAP2B in ESCC [49]. Of note, according to reports, there is a potential connection between RAP2B and the activation of mast cells. Additionally, the presence of mast cells in the esophagus muscularis propria has been established in ESCC patients [49].

Concerning the underlying mechanism, the impact of glucose metabolism on the prognosis and progression of ESCC patients cannot be separated from tumor immunological activities. In the present study, notable differences between the HRG and LRG groups were observed in various immune cell types and immune regulators, including eosinophils and M2 macrophages. The lactate produced by glycolysis can promote the polarization of M2 macrophages, which can facilitate the growth of tumors [50]. It is suggested that infiltrated M2 macrophages could promote the migration and invasion of ESCC cells by facilitating epithelial–mesenchymal transition [51]. Being part of the immune microenvironment, eosinophils play a role in preventing tumor development in several cancers [52, 53, 54], which are involved in antitumor immune responses through multiple mechanisms, encompassing intricate cross‐talk with other tumor cells [55]. Interestingly, the role of eosinophils in the TME may be dual faceted, potentially contributing to both the enhancement of antitumor immunity and, in certain instances, the facilitation of tumor growth [56]. In the subsequent enrichment analysis, immune‐related biological functions, including the cytosolic DNA‐sensing pathway [57] and the mTORC1 signaling pathway [58], exhibited significant enrichment. There is an association between cytokine secretion and cytosolic DNA sensing, and it is essential for tumor control [59]. To be specific, cytosolic DNA sensing triggers the release of immunomodulatory cytokines through transcriptional and posttranslational signaling modules, which is a promising approach to cancer immunotherapy [60, 61]. It has been demonstrated that mTORC1 signaling pathway plays a crucial role in promoting the progression of ESCC [62]. Investigating from a single‐cell perspective, it is the first time we observed that plasma cells have been shown to express higher levels of SERP1 and SSR4 than the other 12 kinds of cells, and CTSC was highly expressed in macrophages. Currently, there is no literature elaborating on how these four hub genes directly regulate the cancer microenvironment in ESCC. Based on these findings, we speculate that the four hub genes may help accentuate the pivotal role in the pathogenesis of ESCC, especially in the dysregulation of the tumor immune microenvironment.

Next, drug sensitivity analyses were applied between the HRG and LRG employing the GDSC database. Compared with HRG, LRG patients exhibited high drug sensitivity on tyrosine kinase inhibitor (axitinib) [63], polyadenosine diphosphate‐ribose polymerase‐1 inhibitors (AG.014699 and AZD.2281) [64, 65], Bcl‐2/xL inhibitor (ABT.263) [66], TANK‐binding protein‐1 inhibitor (BX.795) [67], and PP2Cδ inhibitor (CCT007093) [68], which may become potential druggable targets for ESCC patients. At present, some of these drugs have not yet been adopted in clinical practice, so it is necessary to conduct prospective clinical studies and experimental work to further explore which group of patients can benefit more from characterized chemotherapy.

Our study successfully developed, examined, and confirmed a model that incorporates SERP1, CTSC, RAP2B, and SSR4. This model was created by combining scRNA‐seq and bulk RNA‐seq data to predict the prognosis of ESCC based on risk stratification using the calculated glucose metabolic score. Our findings highlight the potential clinical and therapeutic implications of this model for ESCC patients. However, our research does have significant drawbacks. Firstly, this investigation was analyzed using public databases, limited by an inherent case selection bias due to its retrospective nature, which needs to be validated in vivo through future experiments. Secondly, this study is based on transcriptomics; however, a thorough examination would require the integration of spatial information along with in‐depth transcriptomic or proteomic analysis. Thirdly, to affirm the robustness and applicability of the model, it is imperative to gather comprehensive clinical evidence from ESCC patients, as well as patients with different pathological types.

## 5 Conclusion

An integrated analysis of scRNA‐seq data and clinical datasets was performed to identify key prognostic biomarkers and construct a robust predictive model for ESCC. Four hub genes (SERP1, CTSC, RAP2B, and SSR4) screened based on glucose metabolism developed a predictive model in ESCC patients. The RS was established as an independent risk factor for predicting prognosis. These findings may enhance understanding of ESCC's molecular profile and serve as a new prognostic tool for better patient stratification and treatment planning in clinical practice.

## Author Contributions

**Jiaqi Zhang:** conceptualization (equal), data curation (equal), formal analysis (equal), funding acquisition (supporting), methodology (equal), software (lead), validation (lead), writing – original draft (lead), writing – review and editing (equal). **Shunzhe Song:** conceptualization (equal), data curation (lead), formal analysis (equal), investigation (lead), resources (equal), validation (equal), writing – original draft (equal), writing – review and editing (equal). **Yuqing Li:** data curation (equal), formal analysis (equal), investigation (supporting), software (supporting), writing – original draft (supporting), writing – review and editing (equal). **Aixia Gong:** conceptualization (lead), project administration (lead), resources (lead), supervision (lead), visualization (lead), writing – review and editing (lead).

## Ethics Statement

The current investigation adhered to the guidelines outlined in the Helsinki Declaration. Approval for the investigation was granted by the Ethics Committee of the First Affiliated Hospital of Dalian Medical University (No. PJ‐KS‐KY‐2024‐406).

## Conflicts of Interest

The authors declare no conflicts of interest.

## Supporting information


**Figure S1.** Single‐cell preprocessing. (A) Single‐cell quality control shows each sample’s cell number, gene number, and sequencing depth. (B) The left picture is the relationship between cell sequencing depth and mitochondrial content, and the right image is the relationship between sequencing depth and gene number. The two are positively correlated. (C) Genes that differed significantly between cells and plotted characteristic variance. (D, E) Display of PCA and PC distribution; dots represent cells and colors represent samples. (F) Variance ranking plot for each PC.

**Figure S2.** Univariate Cox analysis of RS and clinical correlation analysis. (A‐E) Association of RS and clinical characteristics (gender and TNM stage) of ESCC patients. (F) Cox regression (univariate) manifested that the RS was an autonomous risk factor (*p* < 0.001) in ESCC patients.

**Figure S3.** Assessment of immune infiltration. (A) Relative infiltrating proportion of 22 immune cells in HRG and LRG. (B) Variations in immune cell infiltration between the low‐ and high‐RS groups. (C) Expression pattern of immunostimulatory factors. (*p < 0.05, **p < 0.01, ***p < 0.001). High risk is shown in pink, and low risk in blue.

**Figure S4.** Expression pattern of (A) immunosuppressive factors, (B) MHC genes, (C) chemokine receptors, and (D) chemokines in HRG and LRG. (*p < 0.05, **p < 0.01, ***p < 0.001.) High risk is shown in pink, and low risk in blue.

**Figure S5.** Model of transcriptional regulation of the four genes. (A) The model genes transcription factor regulatory network, while the blue node represents transcription factors and the pink node represents differentially expressed genes. (B) Enrichment analysis of transcription factor‐binding motifs of model genes is depicted.

**Figure S6.** Construction of a nomogram. (A) The nomogram model was created according to the RS and different clinicopathological variables. (B) Calibration of nomogram‐predicted 1‐year OS and 2‐year OS. (C) ROC curve analysis of the nomogram. (D) Decision curve analysis of the nomogram.

## Data Availability

The article materials include the original contributions presented in this study, code for analysis and further inquiries can be available from the corresponding author on reasonable request.
