# Supplementary material for: Predicting Outcomes in Esophageal Squamous Cell Carcinoma Using scRNA‐Seq and Bulk RNA‐Seq: A Model Development and Validation Study
Source: Cancer Med. 2025 Jan 22;14(2):e70617. doi: 10.1002/cam4.70617 (PMC11751878; doi:10.1002/cam4.70617)
Supplement: Supplementary file 1 — Figure S1. Single‐cell preprocessing. (A) Single‐cell quality control shows each sample’s cell number, gene number, and sequencing depth. (B) The left picture is the relationship between cell sequencing depth and mitochondrial content, and the right image is the relationship between sequencing depth and gene number. The two are positively correlated. (C) Genes that differed significantly between cells and plotted characteristic variance. (D, E) Display of PCA and PC distribution; dots represent cells and colors represent samples. (F) Variance ranking plot for each PC. [file CAM4-14-e70617-s002.pdf]

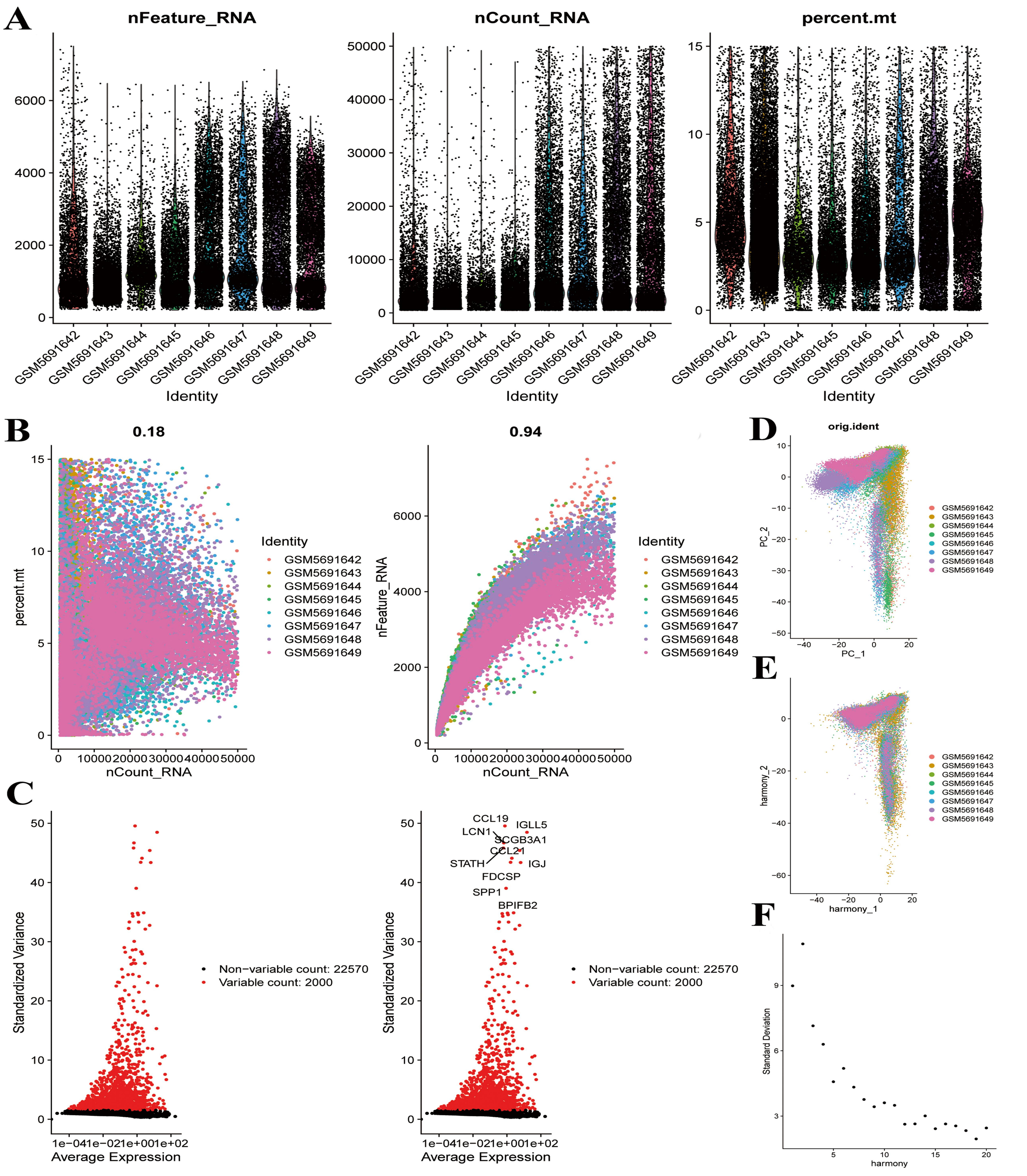

Figure S1 Single cell pre-processing. **(A)** Single-cell quality control shows each sample's cell number, gene number, and sequencing depth. **(B)** The left picture is the relationship between cell sequencing depth and mitochondrial content, and the right image is the relationship between sequencing depth and gene number. The two are positively correlated. **(C)** Genes that differed significantly between cells and plotted characteristic variance. **(D-E)** Display of PCA and PC distribution, dots represent cells, colors represent samples. **(F)** Variance ranking plot for each PC.
