# Supplementary material for: Predicting Outcomes in Esophageal Squamous Cell Carcinoma Using scRNA‐Seq and Bulk RNA‐Seq: A Model Development and Validation Study
Source: Cancer Med. 2025 Jan 22;14(2):e70617. doi: 10.1002/cam4.70617 (PMC11751878; doi:10.1002/cam4.70617)
Supplement: Supplementary file 2 — Figure S2. Univariate Cox analysis of RS and clinical correlation analysis. (A‐E) Association of RS and clinical characteristics (gender and TNM stage) of ESCC patients. (F) Cox regression (univariate) manifested that the RS was an autonomous risk factor (p < 0.001) in ESCC patients. [file CAM4-14-e70617-s006.pdf]

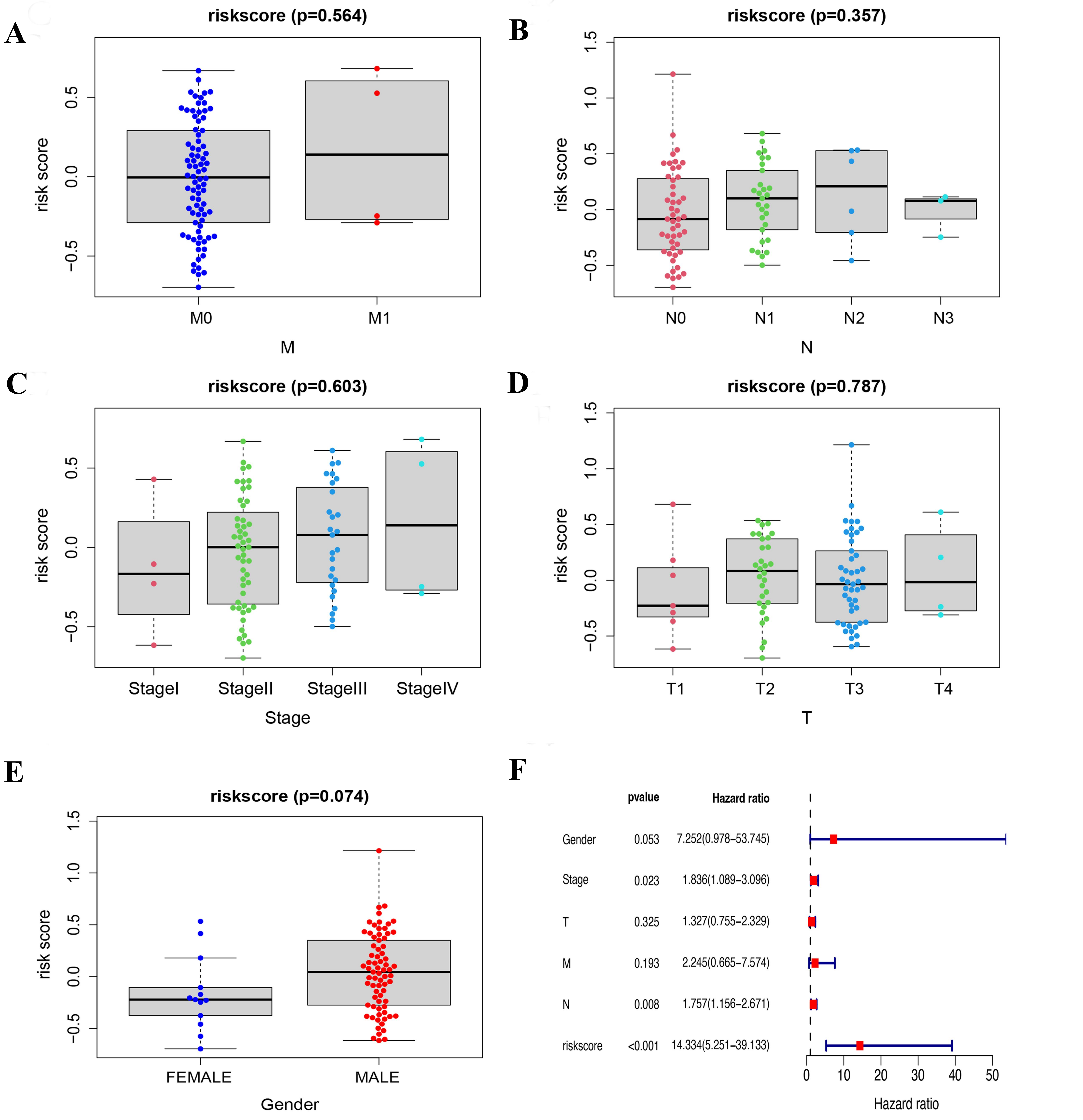

Figure S2 Univariate Cox analysis of RS and clinical correlation analysis. **(A-E)** Association of RS and clinical characteristics (gender and TNM stage) of ESCC patients**. (F)** Cox regression (univariate) manifested that the RS was an autonomous risk factor (*P*<0.001) in ESCC patients.
