# Supplementary figures and images for: Predicting Outcomes in Esophageal Squamous Cell Carcinoma Using scRNA‐Seq and Bulk RNA‐Seq: A Model Development and Validation Study
Source: Cancer Med. 2025 Jan 22;14(2):e70617. doi: 10.1002/cam4.70617 (PMC11751878; doi:10.1002/cam4.70617)

# B

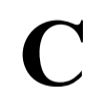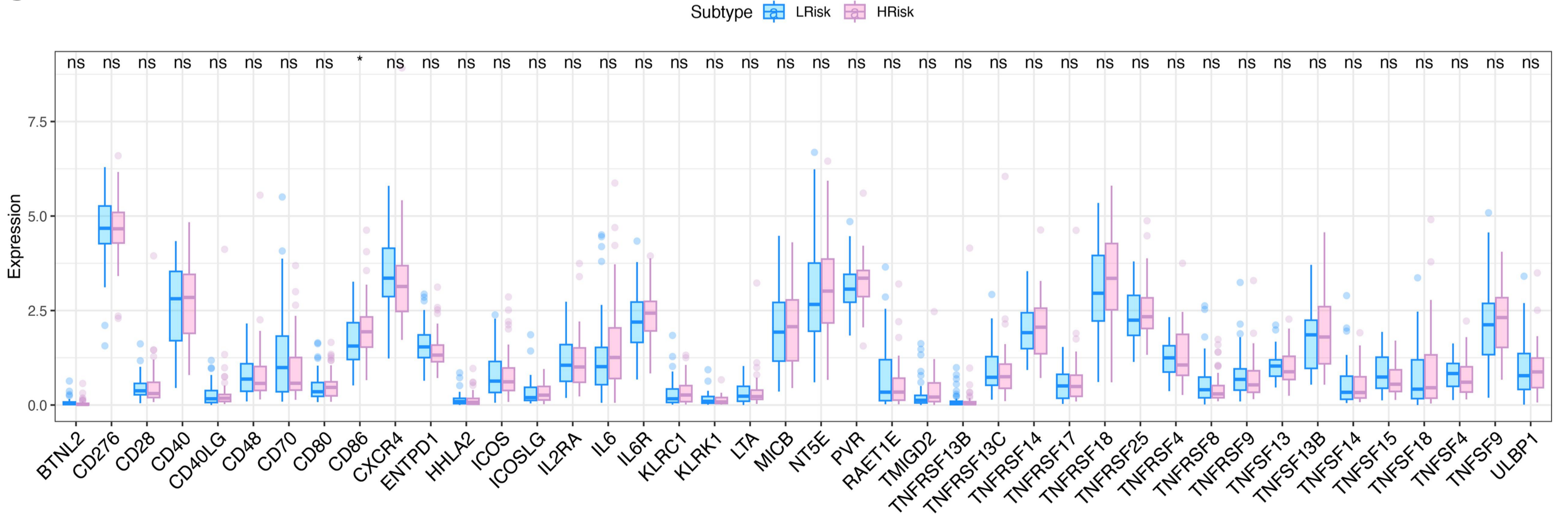

Supplement: Supplementary file 3 — Figure S3. Assessment of immune infiltration. (A) Relative infiltrating proportion of 22 immune cells in HRG and LRG. (B) Variations in immune cell infiltration between the low‐ and high‐RS groups. (C) Expression pattern of immunostimulatory factors. (*p < 0.05, **p < 0.01, ***p < 0.001). High risk is shown in pink, and low risk in blue. [file CAM4-14-e70617-s005.pdf]
