# Supplementary material for: Predicting Outcomes in Esophageal Squamous Cell Carcinoma Using scRNA‐Seq and Bulk RNA‐Seq: A Model Development and Validation Study
Source: Cancer Med. 2025 Jan 22;14(2):e70617. doi: 10.1002/cam4.70617 (PMC11751878; doi:10.1002/cam4.70617)
Supplement: Supplementary file 4 — Figure S4. Expression pattern of (A) immunosuppressive factors, (B) MHC genes, (C) chemokine receptors, and (D) chemokines in HRG and LRG. (*p < 0.05, **p < 0.01, ***p < 0.001.) High risk is shown in pink, and low risk in blue. [file CAM4-14-e70617-s001.pdf]

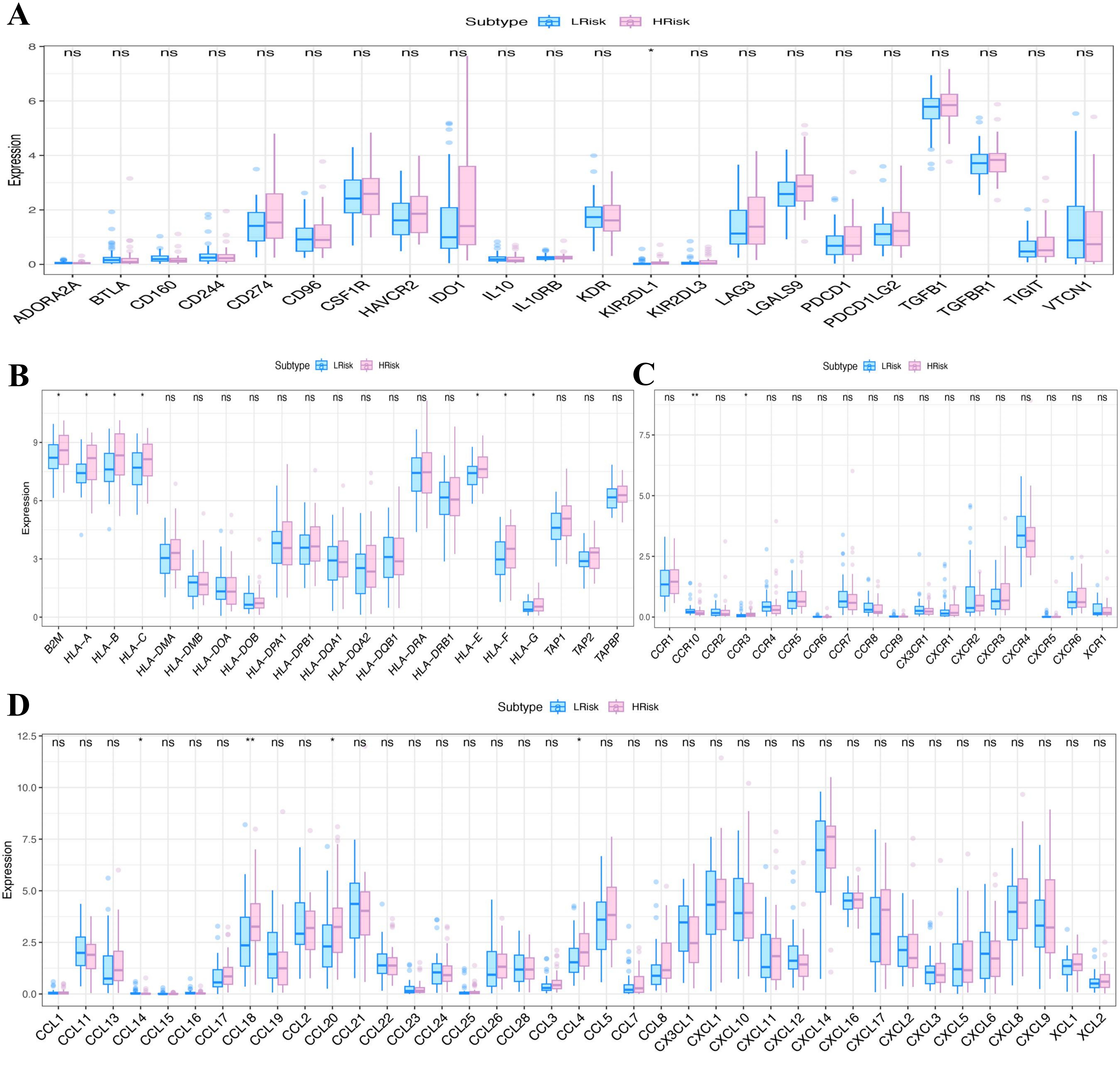


**Figure S4** Expression pattern of **(A)** immunosuppressive factors, **(B)** MHC genes, **(C)** chemokine receptors, and **(D)** chemokines in HRG and LRG. (**P*<0.05, ***P*<0.01, ****P*<0.001) High risk is shown in pink, and low risk in blue.
