# Supplementary material for: Predicting Outcomes in Esophageal Squamous Cell Carcinoma Using scRNA‐Seq and Bulk RNA‐Seq: A Model Development and Validation Study
Source: Cancer Med. 2025 Jan 22;14(2):e70617. doi: 10.1002/cam4.70617 (PMC11751878; doi:10.1002/cam4.70617)
Supplement: Supplementary file 5 — Figure S5. Model of transcriptional regulation of the four genes. (A) The model genes transcription factor regulatory network, while the blue node represents transcription factors and the pink node represents differentially expressed genes. (B) Enrichment analysis of transcription factor‐binding motifs of model genes is depicted. [file CAM4-14-e70617-s003.pdf]

A

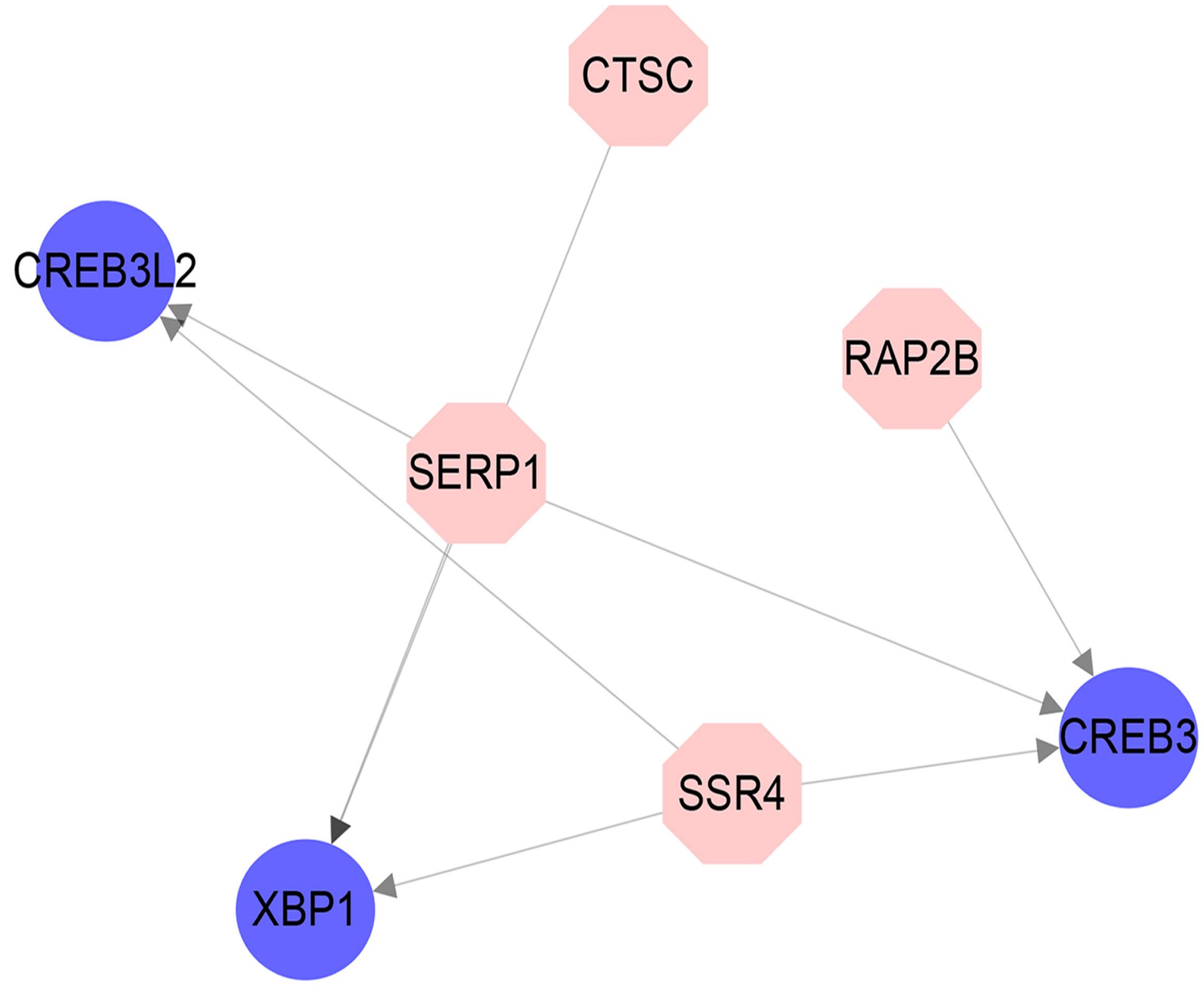

B

	logo	geneSet	motif	NES	AUC	TF_highConf	nEnrGenes	enrichedGenes
	All	All	All	All	All	All	All	All
1	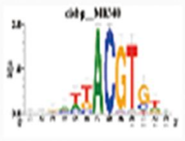	key_gene	cisbp_M0340	7.84	0.461		2	SERP1;SSR4
2	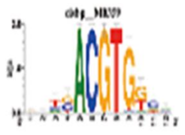	key_gene	cisbp_M0359	7.07	0.417		3	RAP2B;SERP1;SSR4
3	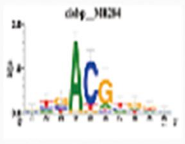	key_gene	cisbp_M0284	6.99	0.413		2	SERP1;SSR4
4	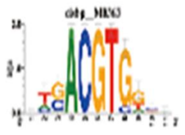	key_gene	cisbp_M0363	6.84	0.404		2	SERP1;SSR4
5	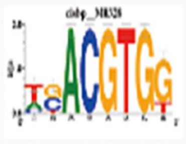	key_gene	cisbp_M0328	6.73	0.398		3	RAP2B;SERP1;SSR4

Figure S5 Model of transcriptional regulation of the four genes. **(A)** The model genes-transcription factor regulatory network, while the blue node represents transcription factors and the pink node represents differentially expressed genes.**(B)** Enrichment analysis of transcription factor binding motifs of model genes is depicted.
