# Supplementary material for: Predicting Outcomes in Esophageal Squamous Cell Carcinoma Using scRNA‐Seq and Bulk RNA‐Seq: A Model Development and Validation Study
Source: Cancer Med. 2025 Jan 22;14(2):e70617. doi: 10.1002/cam4.70617 (PMC11751878; doi:10.1002/cam4.70617)
Supplement: Supplementary file 6 — Figure S6. Construction of a nomogram. (A) The nomogram model was created according to the RS and different clinicopathological variables. (B) Calibration of nomogram‐predicted 1‐year OS and 2‐year OS. (C) ROC curve analysis of the nomogram. (D) Decision curve analysis of the nomogram. [file CAM4-14-e70617-s004.pdf]

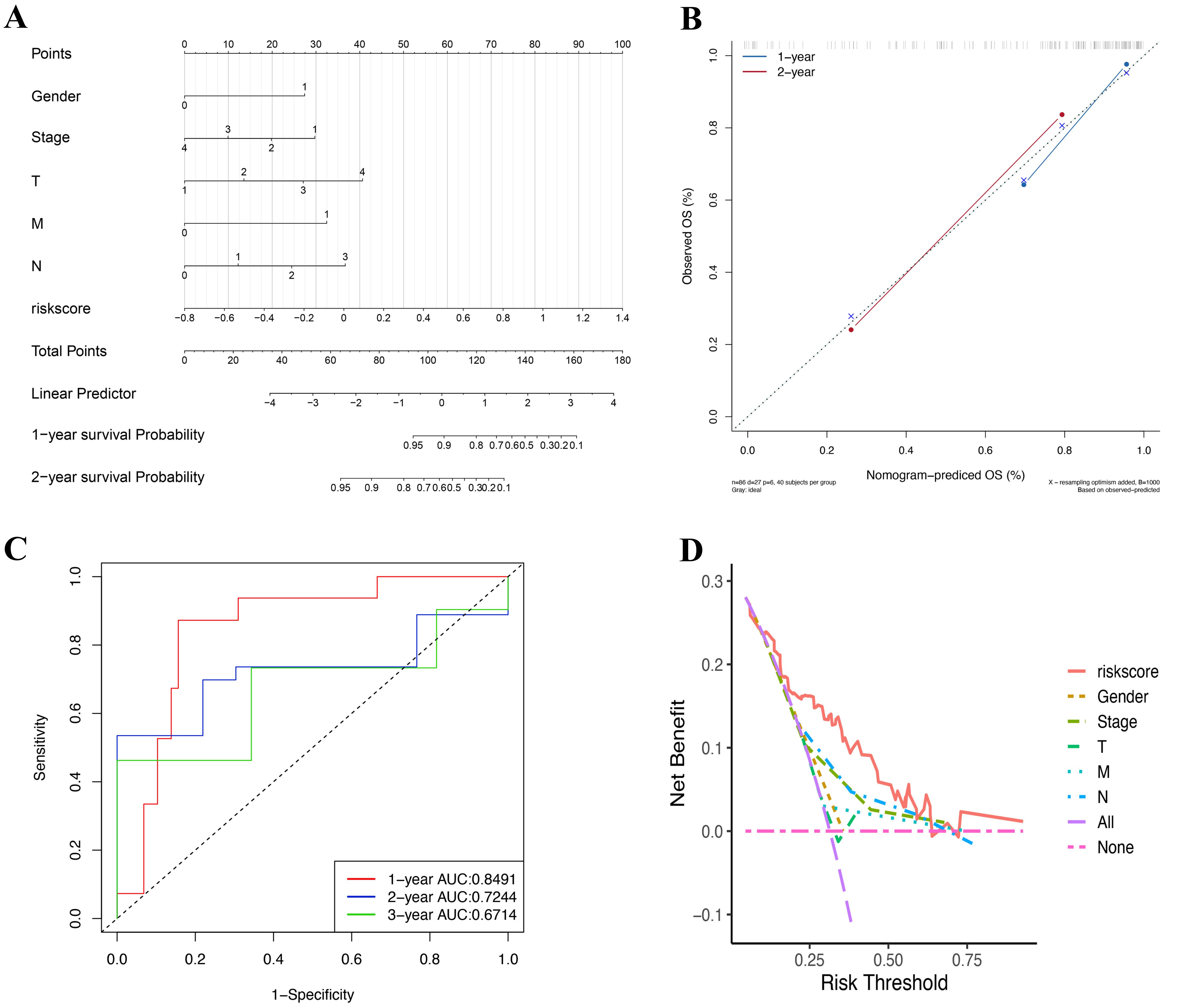


**Figure S6** Construction of a nomogram. **(A)** The nomogram model was created according to the RS and different clinicopathological variables. **(B)** Calibration of nomogram-predicted 1- OS and 2-year OS. **(C)** ROC curve analysis of the nomogram. **(D)** Decision curve analysis of the nomogram.
